# Responses of soil and rhizosphere microbial communities to Cd-hyperaccumulating willows and Cd contamination

**DOI:** 10.1186/s12870-024-05118-0

**Published:** 2024-05-14

**Authors:** Jie Zhou, RuiQing Zhang, Pu Wang, Yunpeng Gao, Jue Zhang

**Affiliations:** 1 Jiangsu Academy of Forestry, Nanjing, China; 2 Jiangsu Suqian Environmental Monitoring Center, Suqian, China; 3 Nanjing Forestry University, Nanjing, China

**Keywords:** Bacterial community, Bulk soil, CO_2_ fixation, Fungal community, High-accumulating willow, N metabolism, Rhizosphere microbes

## Abstract

**Background:**

The pollution of soil by heavy metals, particularly Cd, is constitutes a critical international environmental concern. Willow species are renowned for their efficacy in the phytoremediation of heavy metals owing to their high Cd absorption rate and rapid growth. However, the mechanisms underlying microbial regulation for high- and low-accumulating willow species remain poorly understood. Therefore, we investigated the responses of soil and rhizosphere microbial communities to high- and low-Cd-accumulating willows and Cd contamination. We analyzed soil properties were analyzed in bulk soil (SM) and rhizosphere soil (RM) planted with high-accumulating (H) and low-accumulating (L) willow species.

**Results:**

Rhizosphere soil for different willow species had more NH^4+^ than that of bulk soil, and RM-H soil had more than RM-L had. The available phosphorus content was greater in hyper-accumulated species than it was in lower-accumulated species, especially in RM-H. Genome sequencing of bacterial and fungal communities showed that RM-L exhibited the highest bacterial diversity, whereas RM-H displayed the greatest richness than the other groups. SM-L exhibited the highest diversity and richness of fungal communities. *Ralstonia* emerged as the predominant bacterium in RM-H, whereas *Basidiomycota* and *Cercozoa* were the most enriched fungi in SM-H. Annotation of the N and C metabolism pathways revealed differential patterns: expression levels of *NRT2*, *NarB*, *nirA*, *nirD*, *nrfA*, and *nosZ* were highest in RM-H, demonstrating the effects of NO_3_^-^and N on the high accumulation of Cd in RM-H. The annotated genes associated with C metabolism indicated a preference for the tricarboxylic pathway in RM-H, whereas the hydroxypropionate-hydroxybutyrate cycle was implicated in C sequestration in SM-L.

**Conclusions:**

These contribute to elucidation of the mechanism underlying high Cd accumulation in willows, particularly in respect of the roles of microbes and N and C utilization. This will provide valuable insights for repairing polluted soil using N and employing organic acids to improve heavy metal remediation efficiency.

**Supplementary Information:**

The online version contains supplementary material available at 10.1186/s12870-024-05118-0.

## Background

Heavy metals constitute a class of ubiquitously-derived and highly harmful, persistent pollutants, including lead (Pb), cadmium (Cd), chromium (Cr), and hydrargyrum (Hg). The rapid progression of urbanization and industrialization has resulted in a substantial influx of heavy metals elements into the soil ecosystem, thereby exacerbating the prevalence of severe heavy metal pollution in the soil [1]. Cadmium, characterized as a harmful, non-essential nutrient element for plants, generally exists in nature in the form of divalent cations. Global emissions of Cd surpassed 10 million tons in the year 2005, with 82–94% of this emitted Cd infiltrating the soil [2]. Therefore, soil pollution by Cd has become a pressing global concern. Cd pollution exhibits extensive geographical distribution and strong toxicity, and Cd readily undergoes absorption and accumulation by plants, thereby imparting severe repercussions on organisms within affected ecosystems [3]. Over the last decade, the phytoremediation of heavy metal-contaminated soil has garnered attention, with the efficient restoration of Cd-contaminated soil representing a research hotspot in the field of phytoremediation [4]. However, currently known Cd-enriched plants are herbaceous plants characterized by limited biomass, slow remediation efficiency, and difficult recovery, consequently imposing numerous limitations.

The term willow collectively encompasses *Salicaceae* (*Salix* L.) and *Chosenia* (*Chosenia arbutifolia*), which are the fastest-growing tree species in temperate zones. Willows are acknowledged as a preeminent ecological restoration tree species worldwide, characterized by well-developed root systems, large total root volume, large microbial attachment space, and strong resistance coupled with absorption capabilities for both nutrients and various heavy metal ions, such as Cd. Rapid growth and a high degree of resource utilization in willows have an important role in soil pollution remediation [5]. Extensive research has been conducted on the germplasm screening of heavy metals, such as Pb and Cd, in willow trees, including ‘*Salix Jiangsunesis* 172’, *S. integra* ‘Yizhibi’, and ‘Huishan Lake’. Utilizing hydroponic and soil culture methods, enrichment has been demonstrated to manifest predominantly in the roots rather than in the stems and leaves. Furthermore, planting *Salix viminalis* for 6 years was observed to reduce Cd content to 0.4 µg·kg^− 1^ [6], compared to *Thlaspi arvense*, which required 12 years [7]. Previous studies have confirmed that *Salix mybeana* ‘*Salix Jiangsunesis* 2252’ is a high-Cd-accumulating willow species [8]. However, the interactions between microbial communities and high-accumulating willow species are vague. Through short-cycle, dwarf forest operations, willows can harness biomass for energy production while concurrently engaging in phytoremediation, offering a promising approach to effective alleviation of environmental pollution and the addressing of energy crises.

The accumulation of heavy metals in soil affects microbial community structure and enzyme activity in plant roots. It also affects microbial community structure and biodiversity, which depend on the types of heavy metals and microorganisms. Heavy metals can have bidirectional effects on microorganisms, heavy metals have toxic effects on microorganisms, but microorganisms have adsorption effects on heavy metals. Many researchers have studied soil microorganisms under heavy metal conditions for heavy metal remediation [9]. Heavy metal pollution exerts a multifaceted impact on the size, composition, structure, function, and activity of soil bacterial and fungal communities [10]. Heavy metals can induce alterations in the abundance and diversity of microorganisms [11]. Microorganisms can increase plant uptake and the accumulation of heavy metals to enhance phytoextraction efficiency [12]. Soil microbes can affect the mobility and bioavailability of heavy metals by solubilizing metal phosphates, releasing chelating agents, inducing redox changes, and causing acidification [13–18]. Certain microorganisms, such as *Bacteroidota*, *Proteobacteria*, and *Pontibacter*, can enrich and precipitate heavy metals [19, 20]. In canola biomass, *Pseudomonas fluorescens* and *P. tolaasii* inoculations resulted in a substantial improvement in Cd phytoextraction by 72% and 107%, respectively [21]. Moreover, the combination of *Enterobacter* and *Comamonas* strains stimulated Cd immobilization [22]. The advent of sequencing technology has facilitated the identification of an increasing number of functional microorganisms.

The fast growth rate of willow and its high cadmium absorption rate in soil remediation confer it with a wide range of applications. However, the regulatory mechanisms of soil microorganisms in willow and heavy metals remain unclear. Therefore, we analyzed the response of soil and rhizosphere microbial communities to high- and low-Cd-enrichment willow in the context of Cd pollution. We aimed to analyze the diversity of rhizosphere and bulk microbial communities, encompassing bacteria and fungi, in association with high- and low-Cd-accumulating willows using high-throughput sequencing and correlation analyses. Furthermore, we analyzed rhizosphere microorganisms and their interaction with roots for different willow species. These results will advance the understanding of the mechanisms that contribute to the enhanced accumulation of Cd in high-accumulating willows.

## Methods

### The materials and soil collection

The employed willow species were *Salix Jiangsunesis* 2252 and *S. Jiangsunesis* 2011, cultivated in Cd-polluted soil since 2020, with three replicates established in Changshu Jiangsu Province. Determination of eight available elements in soil was performed according to Standard HJ 804–2016. This standard specifies the determination of Cd content in soil by extraction of diethylenetriamine pentaacetic acid (DTPA). *Salix Jiangsunesis* 2251 is a Cd hyper-accumulated species. It accumulates Cd up to 250 mg.kg^− 1^ in the leaf and 64.55 mg.kg^− 1^ in the stem. The bioconcentration factor (BCF) and translocation factor (TF) in the stem were 54.34 and 6.19 in the stem, respectively. The lower accumulator willow, ‘*Salix Jiangsunesis 2011’*, accumulated Cd at 21.71, 29.19, and 9.69 mg.kg^− 1^ in the leaf, stem, and root, respectively. The BCF and TF were 3.4 and 5.69, respectively. The hyper- and low- accumulated willows were randomly planted with six replicates in the field. The soil collected in April 2022 and each replicate had 15 seedlings in a 40 × 60 cm^2^ row spacing area. The bulk soil for high (SM-H) and low-accumulating willows (SM-L) was collected from the 0–20 cm soil layer in the plot test. The bulk soil was collected from each plot planted with willows and mixed into a composite sample. The rhizosphere soil was collected from the 1 mm surface of soil tightly adhering to the root [1, 5, 9]. Both bulk and rhizosphere soil samples for high- and low-accumulating willows were collected and stored at -4 °C, with six replicates each.

### Analysis of soil physical and chemical properties

The collected soil samples were homogenized, air-dried, and sieved to a particle size of 2 mm. Standard pedological analysis methods were employed to determine soil properties at each site. The control samples were collected from five different locations without willows planting from the 0–20 cm soil layer and mixed well to detect the soil properties as CK. All soil samples were synchronously analyzed for chemical properties and DNA extraction [1]. The samples from hyper-accumulated, and low-accumulated willows in bulk and rhizosphere soil were defined as SM-H, SM-L, RM-H, and RM-L, respectively. The total N content was determined by using a NY-T 2419 − 2013 automatic nitrogen analyzer. Determination of total phosphorus in soil was accomplished by performing composite acid solution/inductively coupled plasma emission spectrometry. The standard TNAIA 70-2021 was used to detect the total carbon content of plants.

### Bacterial and fungal sequencing

Following the extraction of genomic DNA from the samples, the conserved regions of rDNA were amplified using barcode-specific primers, and PCR amplification products were quantified using a QuantiFluor™ fluorometer. The samples were transported to Shanghai Biozeron Biotechnology Co., Ltd for Bacterial and fungal sequencing. The purified amplification products were mixed in equal amounts, and a sequencing library was constructed and subsequently sequenced using the Illumina PE250. After obtaining raw reads, DADA2 (Divisive Amplicon Denoising Algorithm) software [23] was used for data filtering and quality control. The overlapping of pair-end data was conducted, followed by quality control and chimeric filtering to obtain high-quality, clean data. Species annotation, α diversity analysis, β diversity analysis, and community function prediction were performed in sequence according to the analysis process. In the case of valid grouping, a difference comparison and difference test between groups were performed. Finally, combined with other factors (such as environmental factors), specific advanced analyses were performed to explore the relationship between microorganisms and the environment.

### Functional annotation

Based on the sequence information of operational taxonomic units/amplicon sequence variants (OTU/ASV), species annotation serves to correlate analysis results with actual biological significance, facilitating the exploration of relationships between species changes in the community. Representative AVS sequences were obtained using RDP Classifier’s, Naïve Bayesian assignment algorithm, to annotate the species in the database (with a confidence threshold of 0.8–1). KRONA was used to visualize the species annotation results. According to the abundance data of species classification, a heat map was generated to illustrate the expression of different species across varieties. The clustering relationship among species on the heat map reflected the species distribution pattern. The species connection was analyzed using the R language, *psych* package based on species abundance. Pearson correlation coefficients between species were calculated, with Fisher’s-Z transformation employed for statistical testing to obtain P-values. The default criterion involved selecting relationship pairs with a correlation coefficient greater than 0.5. Subsequently, the *igraph* tool was employed to construct a network diagram.

### Statistical analysis

The statistical package SPSS 19.0 for Windows (SPSS Inc., Chicago, IL, USA) was used for all data analyses. Duncan’s new multiple range method was used to determine if differences from multiple comparisons were significant. One-way analysis of variance was used to compare mortality rates, pupation rates in the feeding experiments, and protein content in leaves. Origin 9.0 for Windows (Origin Lab Corp., Northampton, MA, USA) was used to create the graphs.

## Results

### Rhizosphere and bulk soil properties

To investigate soil properties after the cultivation of high- and low-accumulating willows, soil pH, total N (TN), and total C (TC) were assessed. The TN decreased substantially in CK for bulk and rhizosphere soil, with SM-L exhibiting the most substantial decrease. Both NO_3_^−^ and dissolved organic nitrogen (DON) levels were higher in CK and SM-L than in the other samples. NH_4_^+^, dissolved organic carbon (DOC), and available phosphorus (AP) were markedly increased in SM-H. The SM-L group exhibited the highest root water and Cd content (Table 1). In rhizosphere soil, DON, NH_4_^+^, DOC, and AP content were higher in low-accumulating willows than in high-accumulating willows. In bulk soil, NO_3_^−^, DON, DOC, water content, and root Cd content were substantially higher in SM-L than in bulk soil planted with high-accumulating willows (SM-H). No differences were observed in the levels of AK, AP, and NH_4_^+^ in bulk soil compared to those in CK.

**Table 1:** Properties of rhizosphere and bulk soil planted with high- and low-accumulating willows

Type	CK	SM-H	SM-L	RM-H	RM-L
pH	7.32 ± 0.108bc	6.99 ± 0.261b	6.41 ± 0.369a	7.04 ± 0.0282b	7.20 ± 0.041b
Water content	0.2447 ± 0.011a	0.249 ± 0.0282a	0.2775 ± 0.018b	0.2518 ± 0.00103a	0.2427 ± 0.0026a
NH4^+^ (mg/kg)	1.305 ± 0.371a	1.5283 ± 0.578a	1.37 ± 0.350a	3.105 ± 1.079b	1.935 ± 0.505a
NO_3_^−^ (mg/kg)	3.4017 ± 0.768b	2.0883 ± 1.101a	3.46 ± 1.541b	2.527 ± 0.223ab	2.135 ± 0.0791ab
DOC (mg/kg)	45.9533 ± 7.647b	24.427 ± 2.392a	43.687 ± 10.429b	74.153 ± 8.007d	58.95 ± 5.323c
DON (mg/kg)	12.512 ± 1.6341b	8.297 ± 2.156a	11.905 ± 3.081b	7.29 ± 0.824a	6.4 ± 0.584a
AP (mg/kg)	7.8317 ± 1.7294a	9.82 ± 2.909ab	7.595 ± 3.931a	12.465 ± 0.853b	7.785 ± 0.542a
AK (mg/kg)	173.88 ± 198.828a	107.52 ± 16.992a	113.4 ± 30.987a	144.06 ± 7.712a	170.94 ± 14.381a
TC (g/kg)	9.6617 ± 0.55351a	14.43 ± 1.471bc	13.5967 ± 2.227b	13.3667 ± 1.804b	15.712 ± 1.0686c
TN (g/kg)	0.93 ± 0.10863a	1.4067 ± 0.129b	1.33 ± 0.24183b	1.21 ± 0.2155b	1.4183 ± 0.108b
CN (g/kg)	10.475 ± 1.052a	10.27 ± 0.680a	10.27 ± 0.867a	11.1467 ± 0.871a	11.1033 ± 0.712a
Cd (mg/kg)	0.0317 ± 0.024a	0.1133 ± 0.090a	0.2833 ± 0.173b	0.05 ± 0.0063a	0.11 ± 0.054a

*Note* Values represent the means ± standard deviation (SD). SM-L, bulk soil (SM) planted with low-accumulating willow (L); SM-H, bulk soil (SM) planted with high-accumulating willow (H); RM-L, rhizosphere soil (RM) planted with low-accumulating willow (L); RM-H, rhizosphere soil (RM) planted with high-accumulating willow (H); TN, total N; TC, total C; DON, dissolved organic nitrogen; DOC, dissolved organic carbon Different letters in the same column indicate significant differences among means at *p* < 0.05 (one-way analysis of variance [ANOVA])

### Sequence data and microbial diversity

A total of 132,681 bacterial raw reads per sample were obtained, and the bacterial quality sequences were grouped into 102,147 tags with 5362 operational taxonomic units (OTUs). Concurrently, a total of 3,927,142 fungal raw reads were obtained and grouped into 116,412 tags, with an average of 563 OTUs. The diversities of bacteria and fungi were lower in the rhizosphere and bulk soil planted with willows than in the CK. For bacteria, the diversity index was substantially reduced in SM-L, whereas it was reduced in SM-H for fungi (Table 2). For fungi, the Shannon and Chao indices were both higher in low-accumulating willows, indicating a greater fungal richness in this group.

**Table 2:** Community diversity of bacteria and fungi in rhizosphere and bulk soil planted with Cd high- and low-accumulating willows

Type	Group	Shannon (diversity)	Simpson	Chao (richness)	Pielou’s evenness	PD-whole tree
Bacteria	RM-H	10.37	0.99	5569.67	0.83	727.44
	RM-L	10.81	1.00	5540.67	0.87	707.68
	SM-CK	10.95	1.00	5782.67	0.88	738.39
	SM -H	10.44	1.00	5026.33	0.85	664.04
	SM-L	10.13	0.99	4894.33	0.83	645.51
Fungi	RM-H	3.60	0.77	457.33	0.41	399.30
	RM-L	3.64	0.78	575.00	0.40	504.45
	SM -CK	4.90	0.84	734.00	0.51	656.09
	SM -H	3.37	0.66	469.33	0.38	430.39
	SM -L	3.91	0.80	583.67	0.43	514.72

*Note* SM-L, bulk soil (SM) planted with low-accumulating willow (L); SM-H, bulk soil (SM) planted with high-accumulating willow (H); RM-L, rhizosphere soil (RM) planted with low-accumulating willow (L); RM-H, rhizosphere soil (RM) planted with high-accumulating willow (H)

### Microbial community composition and relative abundance

**Fig. 1:**
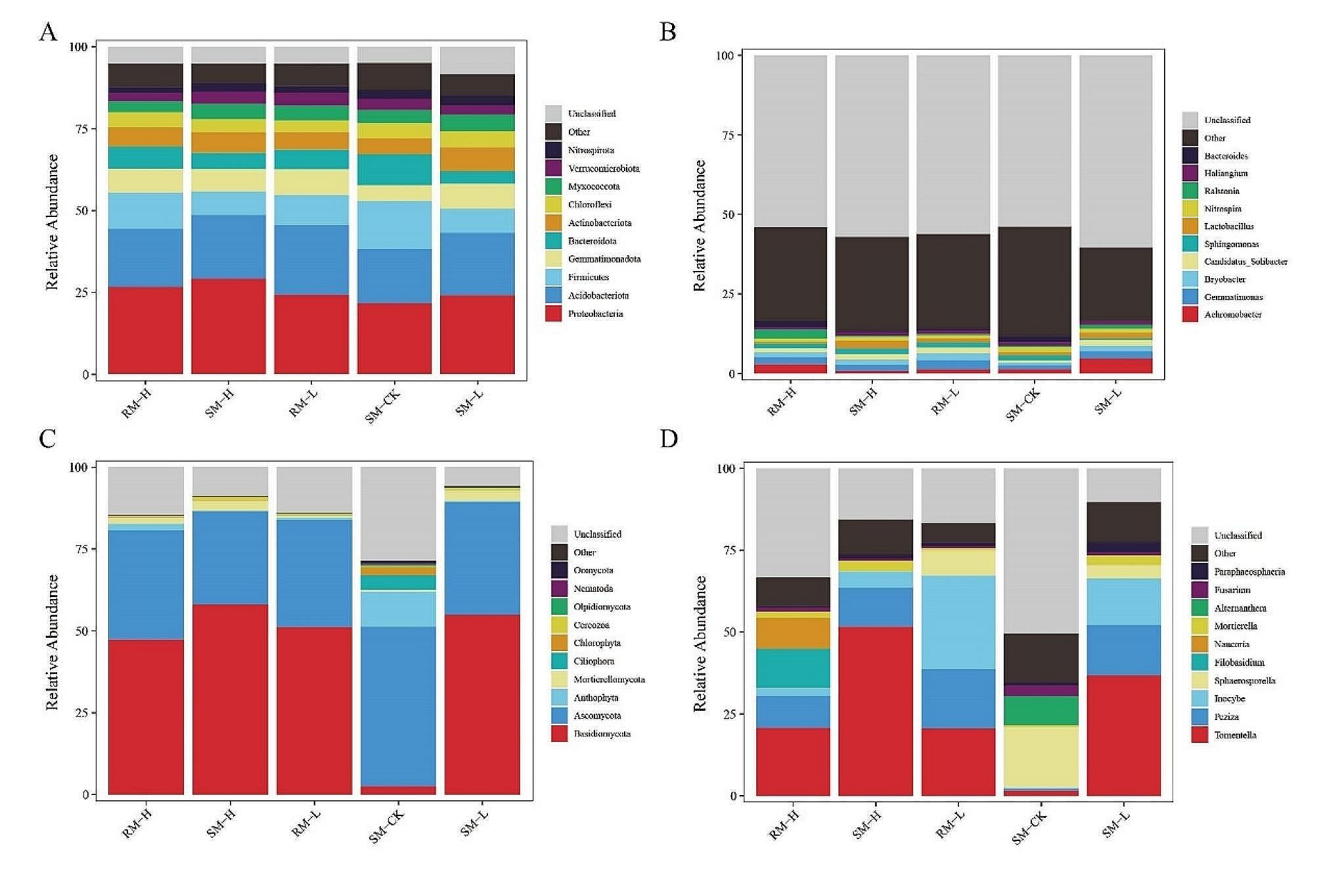
Composition of bacterial communities at the phylum and genus levels and fungal communities at the family and genus levels. (**A**) Relative abundance of bacterial communities at the phylum level. (**B**) Relative abundance of bacterial communities at the genus level. C Relative abundance of fungil communities at the phylum level. D. Relative abundance of fungil communities at the genus level

No statistical differences in bacterial composition were observed at the phylum and genus levels. Proteobacteria and Acidobacteriota exhibited less abundance in SM-CK than in the other soils (Fig. 1A). *Firmicutes* and *Bacteroidota* exhibited more abundance in CK than in the other soils. Proteobacteria were more abundant in rhizosphere and bulk soil planted with Cd high-accumulating willows than in those with low-accumulating willows. At the genus level, *Acidobacteriota*, *Gemmatimonadota*, and *Verrucomicrobiota* were the most abundant bacteria in RM-L, whereas *Ralstonia* was the most predominant in RM-H. *Gemmatimonas* and *Bryobacter* were predominant in RM-L. *Achromobacter* was the most abundant in SM-L. *Lactobacillus* and *Sphingomonas* were more abundant in SM-H than in the other groups (Fig. 1B, D).

Regarding fungal composition, SM-CK exhibited a greater number of components than those of the other groups at both the phylum and genus levels. *Basidiomycota* and *Cercozoa* were the most enriched genera in SM-H. *Mortierellomycota* was the most abundant genus in SM-L. *Anthophyta* and *Mortierellomycota* were more abundant in SM-H than in SM-L. *Basidiomycota* were more abundant in SM-L than in SM-H and CK. At the genus level, the top ten fungal compositions varied. *Naucoria* and *Alternanthera* were absent in RM-L and SM-L, respectively. *Filobasidium* and *Naucoria* were predominant in RM-H, whereas *Tomentella* exhibited higher prevalence in SM-H than in the other groups. *Paraphaeosphaeria* were relatively more abundant in SM-L, whereas *Peziza* and *Inocybe* were more prevalent in RM-H than in the other groups (Fig. 1C, D).

### Functional analysis of microbial communities

**Fig. 2:**
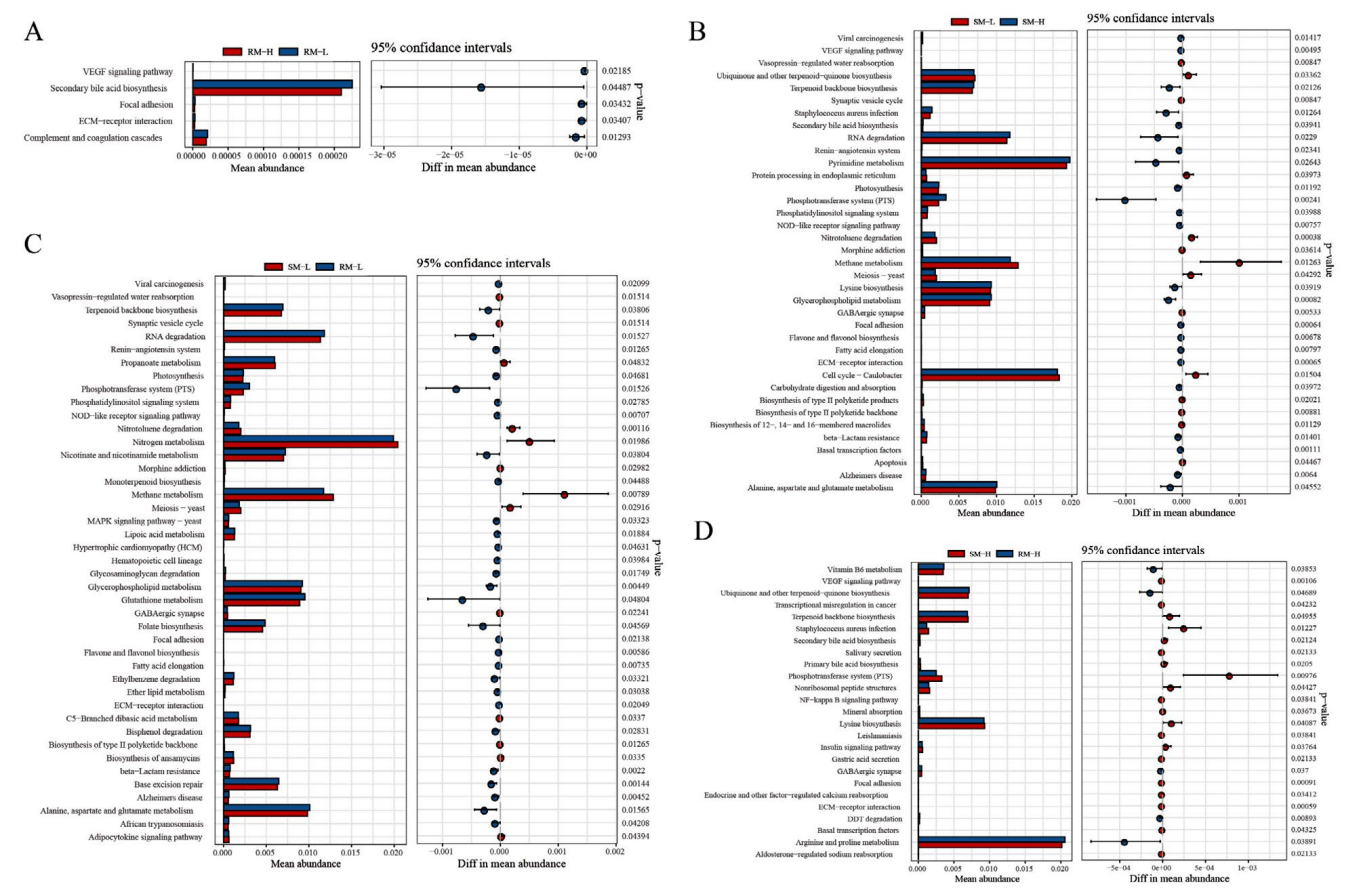
KEGG pathway enriched in microbial communities. KEGG pathway orthology (**A**), RM-H vs. RM-L, (**B**), SM-L vs. SM-H, (**C**), SM-L vs. RM-L, (**D**), SM-H vs. RM-H in bacterial

In the rhizosphere soil, functional genes within the bacterial community were enriched in secondary bile acid biosynthesis and complement and coagulation cascades (Fig. 2). In the bulk soil, pyrimidine metabolism was more abundant in SM-H. Cell cycle and methane metabolism were more enriched in SM-L than in SM-H. For high-accumulating willows, genes were enriched in arginine and proline metabolism, lysine biosynthesis, ubiquinone and other terpenoid − quinone biosynthesis, and terpenoid backbone biosynthesis pathways. Bacterial microbial analysis for low-accumulating willows revealed that genes were more abundant in N metabolism, methane metabolism, alanine, aspartate, and glutamate metabolism, as well as terpenoid backbone biosynthesis. In the sequencing analysis of fungi, higher abundances were observed in SM-L and RM-L than in SM-H, with RM-H exhibiting greater abundance than SM-CK. The predominant functional Kyoto Encyclopedia of Genes and Genomes pathways included wood saprotroph, ectomycorrhizal fungi, dung saprotroph, and bryophytic parasites. Plant parasite was more enriched in RM-H than in other groups.

### Interaction network of RDA analysis

**Fig. 3:**
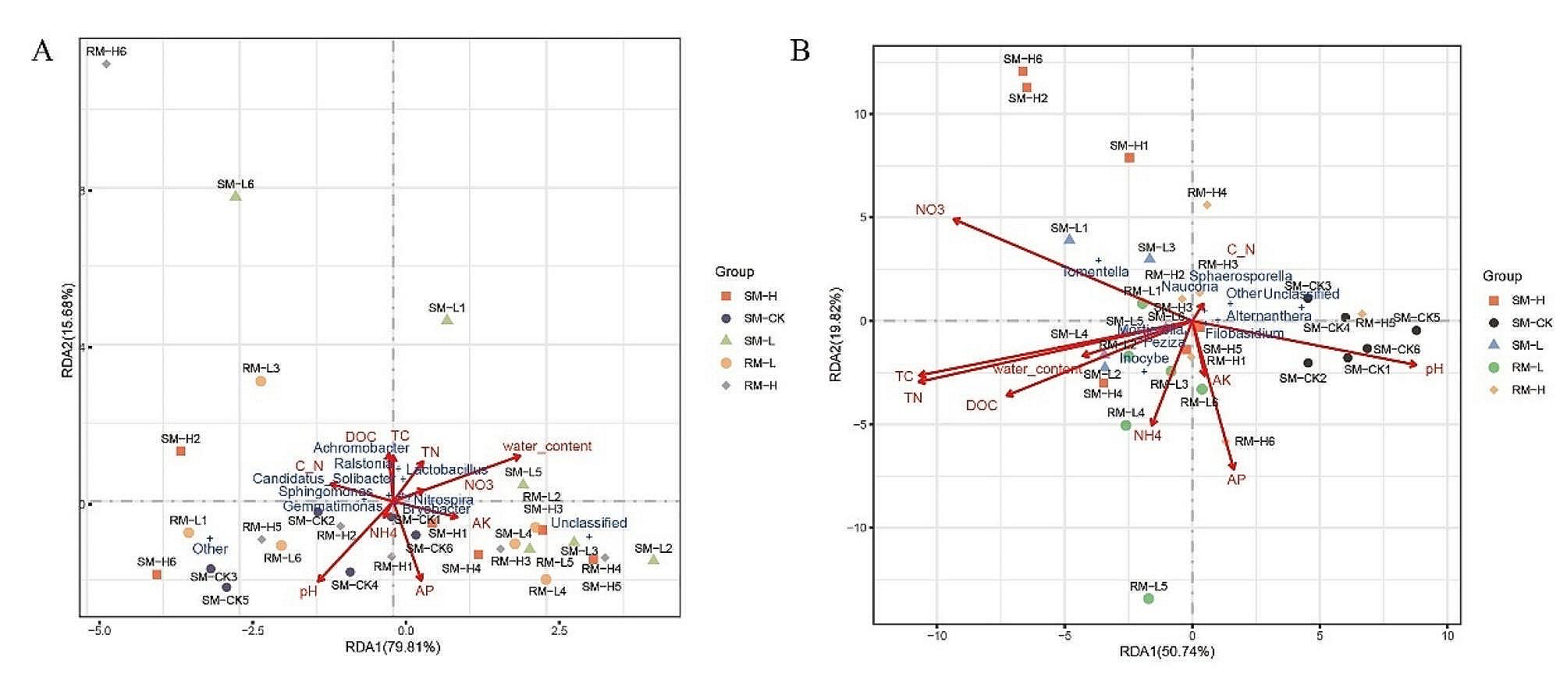
Redundancy analysis to explore the correlations between bacteria (**A**) and Fungi (**B**) functional genes associated with soil P-cycling potential and soil properties in both rhizosphere and bulk soils across grazing intensities. Different colors of dots represent genes extracted from soil samples under different grazing intensities. Arrows represent the influence of different soil physical and chemical properties on functional genes, with arrow length indicating the strength of the influence (explanatory rate)

The interaction network of microbes, soil properties, bacteria, and fungi were generated using redundancy analysis. Water content and pH were highly correlated with the population diversity of bacteria (Fig. 3A), and NO_3_^−^ and pH were highly correlated with the population diversity of fungi (Fig. 3B). In both rhizosphere and bulk soils, the pH was positively correlated with SM-CK. The AP was negatively correlated with RM-L and RM-H. And the NO_3_^−^ was positively correlated with bacterial abundance. For fungi, AP was negative correlated with NO_3_^−^ and exhibited associations with SM-H, SM-L, RM-L, and RM-H (Fig. 3B). There was a high correlation between pH and diversity of bacteria and fungi.

### Nitrogen fixation and carbon metabolism pathways in microbes

**Fig. 4:**
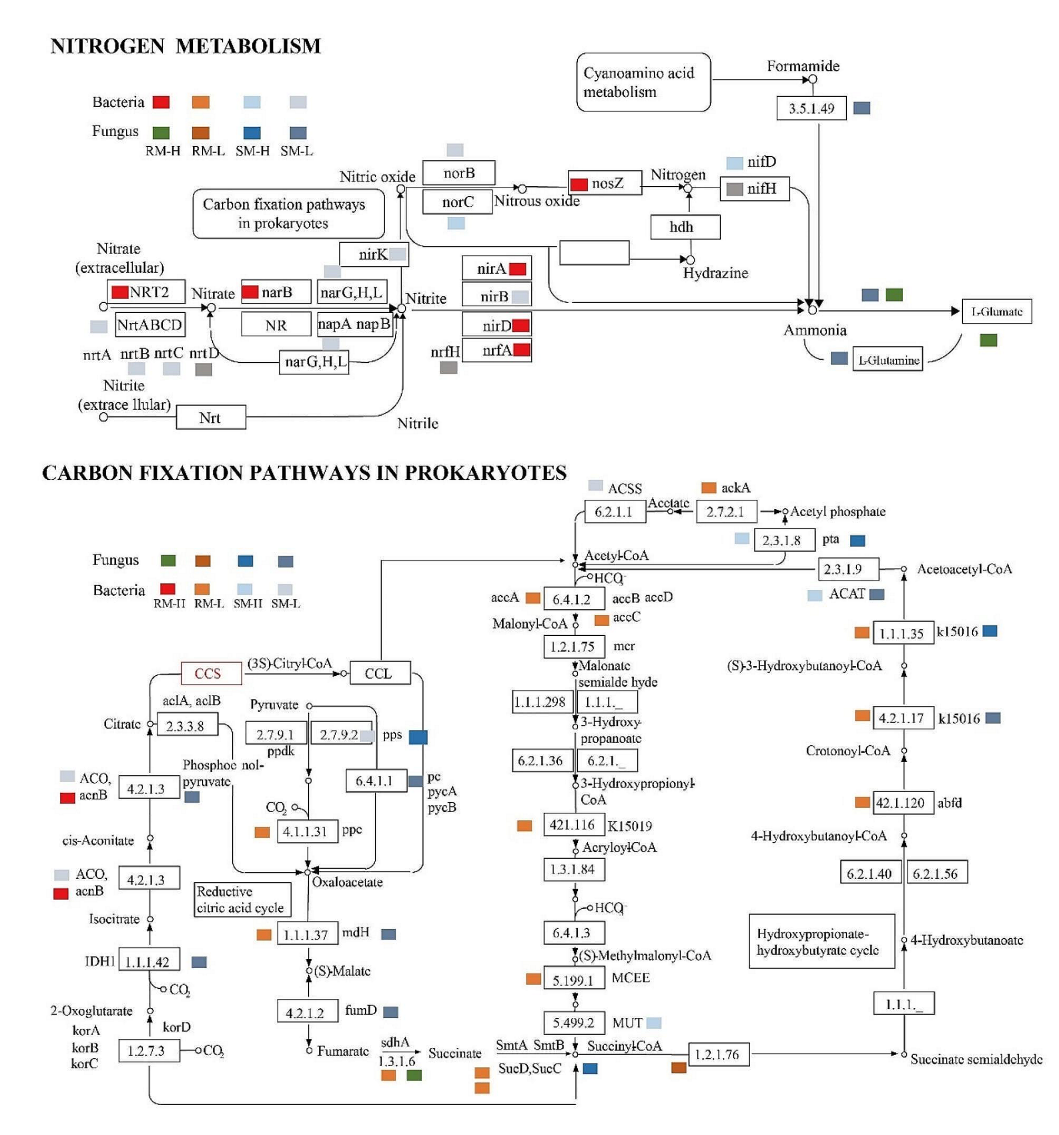
Genes involved in nitrogen fixation and carbon metabolism. *Note*: NRT2, nitrate transporter; narB, nitrate reductase B; narG, nitrate reductase G; narH, nitrate reductase H; narL, nitrate reductase L; norC, nitric oxide reductase subunit C; norB, nitric oxide reductase subunit B; nosZ, nitrous-oxide reductase; nifD, nitrogenase molybdenum-iron protein alpha chain; nifH, nitrogenase iron protein; nirA, nitrogen assimilation transcription factor; nirB, assimilatory sulfite reductase B; nirD, assimilatory sulfite reductase D; nrfA, cytochrome c nitrite reductase subunit A; napA, periplasmic nitrate reductase A; napB, periplasmic nitrate reductase B; nrt, nitrate transporter; aco, aconitate hydratase; acnB, aconitate hydratase 2; korA, 2-oxoacid ferredoxin oxidoreductase subunit alpha; korB, 2-oxoacid ferredoxin oxidoreductase subunit B; korC, 2-oxoacid ferredoxin oxidoreductase subunit C; ppdk, orthophosphate dikinase; ppc, phosphoenolpyruvate carboxylase; pc, pyruvate carboxylase; pycA, pyruvate carboxylase A; pycB, pyruvate carboxylase B; pps, pyruvate, water dikinase; mdH, malate dehydrogenase; fumD, fumarate hydratase; MCEE, methylmalonyl-CoA/ethylmalonyl-CoA epimerase; mcr, malonyl-CoA reductase; SucD, succinyl-CoA synthetase alpha subunit; SmtA, (S)-malate CoA-transferase subunit A; SmtB, (S)-malate CoA-transferase subunit B; SucC, succinyl-CoA synthetase alpha subunit; pta, phosphate acetyltransferase; accA, acetyl-CoA carboxylase biotin carboxyl carrier protein A; accB, acetyl-CoA carboxylase biotin carboxyl carrier protein B; accD, acetyl-CoA carboxylase biotin carboxyl carrier protein D

The bacterial N fixation pathway displayed an enrichment of 22 genes (Fig. 4). Of these, six (*NRT2*, *narB*, *nosZ*, *nirA*, *nirD*, and *nrfA*), two (*norC* and *nifD*), and 11 exhibited the highest expression in RM-H, SM-H, and SM-L, respectively. Concurrently, the bacterial CO_2_ metabolism pathway displayed enrichment of 80 genes. Of these, two (*acnB* and *K15052*) and 19 exhibited the highest expression in RM-H and RM-L, respectively, indicating their role in CO_2_ metabolism in RM-L (Fig. 4).

**Fig. 5:**
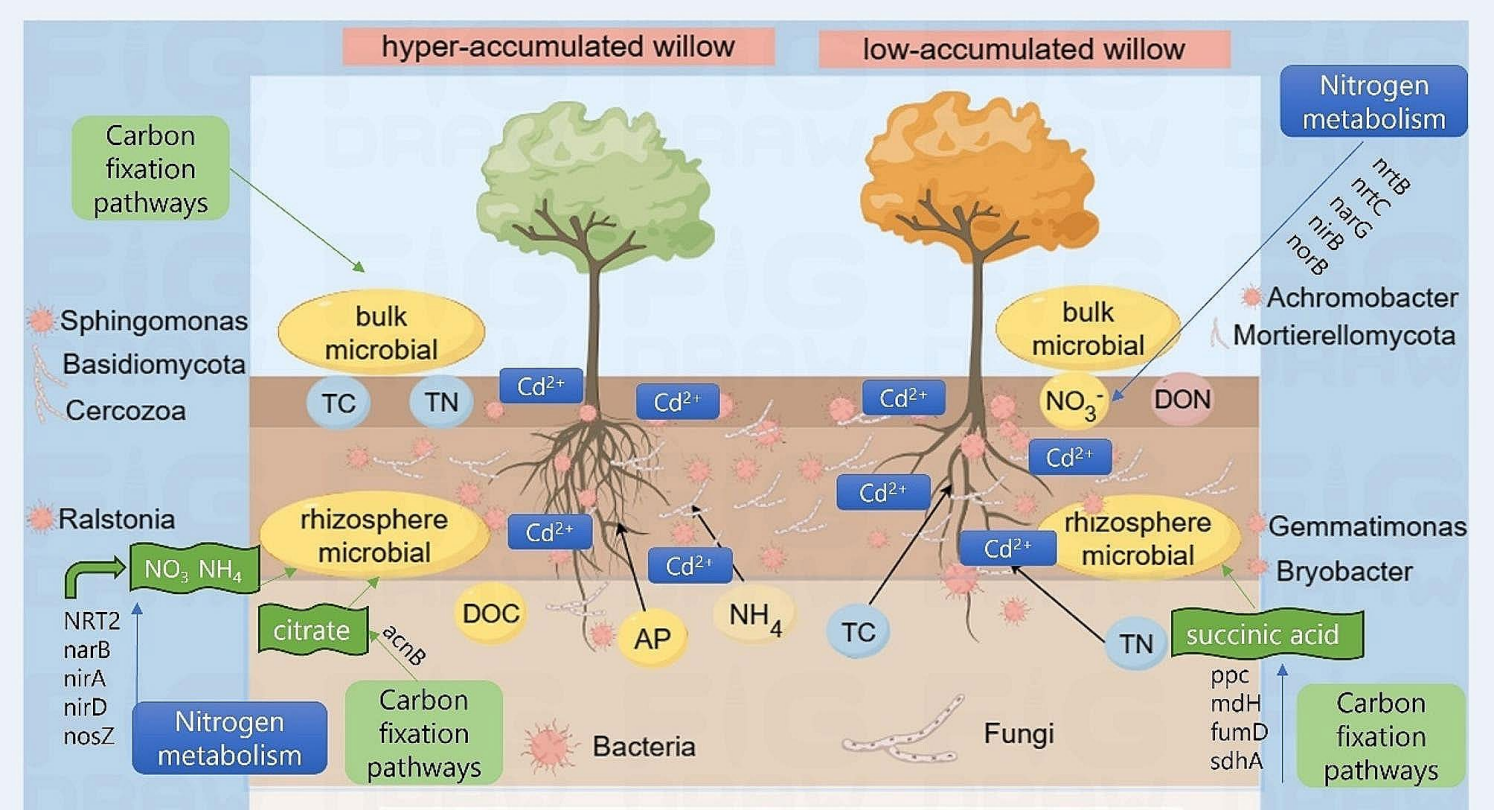
A cartoon model illustrating the proposed microbial regulation network in hyper-accumulated and low-accumulated willows

In the fungal genome, eight genes were annotated in the N metabolism pathway, with EC:1.4.1.3 exhibiting the highest expression in RM-H, four enzymes displaying the highest expression in SM-L, and no genes found to be the most enriched in SM-H. Fourteen enzymes were annotated in the C fixation metabolism pathway, with EC1.3.1.6 being the most prevalent in RM-H (Fig. 4) (Supplementary file 1).

## Discussion

The rhizosphere microbial communities of plants are shaped through intricate interactions with microbes [24]. Different plant species host specific bacterial communities, even when cultivated on the same soil [25]. Soil bacteria have been demonstrated to improve plant growth, crop productivity, and phytoremediation efficiency [26]. Soil structure, composition, and properties undergo substantial transformations in response to heavy metal pollution [27]. The different forms of N resources supplied to plants affect their response to heavy metal and abiotic stress [28]. For instance, NH_4_^+^ improved Cd phytoextraction compared to that of NO_3_^−^ fertilizers in sunflowers, whereas NO_3_^−^ supplementation promoted rice Cd absorption [29]. In *Arabidopsis*, NH_4_^+^ application restricted Cd accumulation and translocation from roots to shoots [30]. In high-accumulating *Solanum nigrum* L., NH_4_^+^ exhibited stronger Cd detoxification ability than that of NO_3_^−^ by decreasing Cd accumulation [31]. As shown in Table 1, SM-H demonstrated substantially higher levels of NH_4_^+^ and AP, whereas SM-L exhibited higher levels of NO_3_^−^. However, no difference was observed between SM-H and SM-L, indicating that high-accumulating willows have a propensity to cluster NH_4_^+^ and utilize NO_3_^−^ in the process of Cd accumulation. These findings suggest that NH_4_^+^ and NO_3_^−^ promoted Cd absorption by willows. Exogenous P has been demonstrated to increase Cd content in rice cell walls [32]. In this study, SM-H exhibited a higher AP content, indicating that P may be stimulated in high-accumulating willows to accumulate more Cd.

The Chao index of bacterial richness was higher for high-accumulating willows, as shown in Table 2, with RM-H exhibiting a higher index than that of SM-H, indicating that increased bacterial richness is favorable for enhanced Cd absorption. Conversely, low-accumulating willows were associated with higher fungal richness, with the index in SM-L lower than that in RM-L. This pattern indicates that fungal enrichment was more prominent in conditions of lower Cd concentration. Regarding diversity, bacterial diversity in RM-L was higher, indicating that bacterial diversity decreased Cd absorption for willows planted in rhizosphere soil. The greater fungal diversities observed in SM-L suggest a positive correlation between fungal diversity and Cd^+^ enrichment.

Bacterial endophytes, isolated from diverse plant species, have demonstrated the potential to promote plant growth or confer higher tolerance to plants cultivated in heavy-metal-contaminated soils [33–37]. Dominant bacterial phyla such as Proteobacteria, Actinobacteria, Firmicutes, and Bacteroidetes have been identified in response to Cd contamination [38]. *Proteobacteria* were predominant in high-accumulating willows, indicating their role in enhancing Cd uptake. Higher AP content and *Proteobacteria* abundance were observed in SM-H and SM-H than in the other soil types, indicating AP and *Proteobacteria* may promote the absorption of cadmium by willow. *Verrucomicrobiota*, a heterotrophic bacterium that utilizes C as a nutrient source, exhibited the highest abundance in RM-L, with the TC content, indicating its function in C utilization. *Ralstonia* has been demonstrated to survive under extremely oligotrophic and heavy metal-polluted environments. *Ralstonia* mixed with biochar increased the adsorption efficiency of Cd by 16.23–40.80% [39]. Furthermore, they can degrade aromatic compounds; therefore, they can be used as bioremediation bacteria [40].

*Ralstonia* accumulation was observed in the rhizosphere of high-accumulating willows, indicating their ability to stimulate Cd absorption. *Achromobacter* were demonstrated to alleviate Cd toxicity [41]. These bacteria exhibited the highest accumulation in SM-L, indicating their detoxification role in minimizing Cd accumulation in willows. *Lactobacillus* is recognized as a Cd-discharge bacteria, and *Sphingomonas* protect *S. alfredii* roots from Cd damage, indicating that *Lactobacillus* and *Sphingomonas* protect high-accumulating willows from Cd toxicity [42]. *Basidiomycota* and *Cercozoa* are important and predominant fungi in most rhizosphere soils, such as those in hybrid rice fields and pine forests [43, 44]. Their dominance in SM-H indicates their resistance to Cd. Conversely, *Mortierellomycota* exhibited a decrease in rhizosphere soils compared to that in bulk soils, possibly owing to their sensitivity to Cd concentration. *Filobasidium* and *Naucoria*, previously unreported to respond to Cd stress, may represent new phytoremediation fungi capable of improving plant Cd accumulation. *Peziza*, recognized for its role in alleviating Cd toxicity in plants, exhibited higher abundance in RM-L, suggesting its effectiveness in reducing willow Cd accumulation [45].

In the bacterial genome of RM-H, the expression of *NRT2*, *narB*, *nirA*, *nirD*, and *nosZ* was observed. These genes represent the core genes involved in N fixation and synthesis from NO_3_^−^ to N and NH_4_^+^. Any form of N can enhance Cd uptake and accumulation in plants [46]. These results indicate that genes enriched in RM-H led to the production of NO_3_^−^, N, and NH_4_^+^, which stimulated Cd hyperaccumulation. Conversely, in low-accumulated willows, the expression of *nrtB*, *nrtC*, *narG*, *nirB*, and *norB* was observed. These genes play a key role in the synthesis of NO_3_^−^, NO_2_, and NO, resulting in higher concentrations of NO_3_^−^ and TN in SM-L. This pattern aligns with the soil property results observed in our study. The elevated content of NH_4_^+^ in RM-L appears to be an important factor that stimulates the root absorption of Cd. In the fungal genome, the presence of genes enriched in L-glutamine in SM-L and L-glumate in RM-H indicate that L-glutamine and L-glumate served as N sources for willows.

For C metabolism, *acnB* was more abundant in RM-H, indicating its role in the tricarboxylic (TCA) cycle. Additionally, *ppc*, *mdH*, *fumD*, *sdhA*, and eight other genes were enriched in the hydroxypropionate-hydroxybutyrate cycle in RM-L, indicating that low accumulators preferred engaging in the TCA and hydroxypropionate-hydroxybutyrate cycles for C sequestration. In the fungal community, most of the highly expressed genes originated from SM-L, suggesting that fungi were more active in bulk soil with low-accumulating willows. AcnB is vital for citrate synthesis in the C metabolism pathway of the TCA cycle. Citrate enhances Cd absorption in wheat [47], stimulates root stability in *Brassicaceae*, and acts in phytoremediation as an organic acid [48]. The high expression observed in RM-H suggests that acnB plays a crucial role in the synthesis of citrate, thereby contributing to the enhanced Cd accumulation in willows. Succinic acid is an important factor for the efficient removal of Cd and has been demonstrated to inhibit Cd uptake and activate Cd outflow in rice [49]. The high expression of the structural genes in RM-L for succinic acid synthesis aligns with this previous observation. This suggests that fumarate and succinic acid play an important role in the bacterial response to Cd toxicity.

In this study, high- and low-accumulation willows demonstrated substantial variations regarding soil properties and microbial diversity, richness, and structure in both rhizosphere and bulk soil. For N metabolism, *NRT2*, *narB*, *nirA*, *nirD*, and *nosZ* led to the production of NO_3_^−^, N, and NH_4_^+^, stimulating Cd hyper-accumulation. Then, *nrtB*, *nrtC*, *narG*, *nirB*, and *norB* emerged as the key genes in the synthesis of NO_3_^−^, NO_2_, and NO, which led to a higher NO_3_^−^ and TN content in SM-L. In the fungal community, L-glutamine in SM-L and L-glumate in RM-H were potential N sources for willows. In the C pathway, citrate enhanced Cd absorption under the regulations of the *acnB* gene in RM-H, whereas succinic acid was identified as detoxification to willows (Fig. 5).

## Conclusions

Willows exhibit important potential for the phytoremediation of heavy metal-polluted soil. We found that RM exhibited the highest bacterial diversity and greater richness than the other groups. SM-L exhibited the highest diversity and richness of fungal communities. Regulation of the microbial C and N metabolism pathways involves different genes, ultimately leading to Cd accumulation and toxicity. This study provides a solid foundation for understanding the mechanisms of willows. A comprehensive understanding of the N and C pathways will provide valuable insights for repairing polluted soil using N and employing organic acids to improve remediation efficiency.

### Electronic supplementary material

Below is the link to the electronic supplementary material.

Supplementary Material 1

## Data Availability

The transcriptome sequence data were submitted to GenBank of NCBI (https://www.ncbi.nlm.nih.gov/) under the accession number: PRJNA1079022 and PRJNA 1079023. The associated BioProject, SRA, and Bio-Sample numbers are as follows: SAMN40028520, SAMN40028521, SAMN40028522, SAMN40028523, SAMN40028524, SAMN40028525, SAMN40028526, SAMN40028527, SAMN40028528, SAMN40028529, SAMN40028530, SAMN40028531, SAMN40028532, SAMN40028533, SAMN40028534, SAMN40028535, SAMN40028536, SAMN40028537, SAMN40028538, SAMN40028539, SAMN40028540, SAMN40028541, SAMN40028542, SAMN40028543, SAMN40028544, SAMN40028545, SAMN40028546, SAMN40028547, SAMN40028548, SAMN40028549.
